# Timing-Dependent Immunostimulatory Activity of Human IFN-β mRNA in Primary Human Myeloid Cells

**DOI:** 10.3390/vaccines14090829

**Published:** 2026-09-20

**Authors:** Silvia Fraude-El Ghazi, Maria José Limeres, Rocio Gambaro, Ana Pena Vaquero, Dirk Prawitt, German Islan, Stephan Gehring, Maximiliano L. Cacicedo

**Affiliations:** Children’s Hospital, University Medical Center of the Johannes Gutenberg University Mainz, Langenbeckstr. 1, 55131 Mainz, Germany

**Keywords:** mRNA therapeutics, IFN-β, cytokine mRNA, dendritic cell activation, macrophage repolarization

## Abstract

**Background/Objectives:** Systemic administration of recombinant cytokines for immunomodulation in cancer and chronic infection is limited by toxicity and poor spatial control. Messenger RNA (mRNA)-encoded cytokines offer a programmable alternative, but the immunological consequences of interferon-β (IFN-β) mRNA delivery in primary human immune cells, and its compatibility with co-delivered antigen-encoding mRNA, remain incompletely defined. This study evaluated whether nucleoside-modified, mRNA-encoded human IFN-β can function as a multifunctional immunomodulator with pro-apoptotic activity and timing-dependent immunostimulatory properties. **Methods:** N1-methylpseudouridine-modified IFN-β mRNA was transfected into HEK293T and HepG2 cell lines and into primary human monocyte-derived dendritic cells (MDDCs) and M2-polarized macrophages (MDMs) using Lipofectamine MessengerMAX, with OVA mRNA as a non-adjuvant control. Apoptosis, interferon-stimulated CXCL10 secretion, activation marker expression, and cytokine profiles were assessed by flow cytometry, ELISA, Western blot, and cytometric bead array. Adjuvant activity was evaluated in MDDCs co-transfected with IFN-β and antigen (EGFP or OVA) mRNA under four temporal delivery regimens. **Results:** IFN-β mRNA induced time-dependent apoptosis, most pronounced in HepG2 cells, and CXCL10 secretion in both cell lines. In primary immune cells, IFN-β mRNA activated MDDCs, increased pro-inflammatory cytokine secretion, and repolarized M2-like macrophages toward a pro-inflammatory phenotype. In co-transfection experiments, IFN-β mRNA enhanced MDDC activation across regimens, but antigen expression was preserved only when antigen mRNA was delivered before IFN-β mRNA. **Conclusions:** mRNA-encoded IFN-β combines direct pro-apoptotic activity with programmable, timing-dependent immunostimulatory effects, supporting its development as a component of next-generation mRNA-based immunotherapies and vaccines.

## 1. Introduction

The immune microenvironment is a critical determinant of outcomes in both malignant and infectious diseases. In cancer, the tumor microenvironment (TME) is a complex network of immune, stromal, and vascular components that strongly influences tumor development, progression, metastasis, and response to therapy. These cellular and molecular networks can promote angiogenesis, immune suppression, and tumor invasion, thereby shielding tumors from effective immune surveillance [1,2,3,4,5,6]. Similarly, during infection, the local immune milieu shapes pathogen clearance, immune activation, and the development of protective immunity [7,8]. Strategies capable of modulating these local immune environments therefore represent an important therapeutic avenue.

mRNA-based therapeutics have emerged as a versatile platform for both vaccination and immune modulation. Beyond enabling the expression of antigenic proteins, mRNA can be engineered to encode immunomodulatory molecules such as cytokines, chemokines, or co-stimulatory ligands [9]. This allows mRNA formulations to combine antigen expression with active shaping of the immune response. Such programmable immunomodulation has broad implications across therapeutic areas, including cancer immunotherapy and vaccines against infectious diseases. In oncology, cytokine-encoding mRNA therapeutics may help overcome immunosuppressive tumor microenvironments, whereas in infectious-disease vaccination they may enhance innate immune activation and promote robust adaptive immunity [10,11]. Importantly, the delivery vehicle itself can also contribute to the immunostimulatory properties of mRNA formulations. In particular, lipid nanoparticles have been shown to induce innate inflammatory responses, including the production of pro-inflammatory cytokines and chemokines [12]. Thus, the overall immunological activity of an mRNA formulation may reflect both the intrinsic effects of the delivery system and the biological activity of the encoded protein.

Among immunomodulatory cytokines, IFN-β was selected for the present study because of its pleiotropic effects on both tumor and immune cells. Its ability to exert tumor-cell-intrinsic effects while simultaneously promoting antigen-presenting-cell activation and modulating myeloid-cell phenotypes makes IFN-β particularly attractive for investigating cytokine-encoding mRNA as an immunomodulatory component of mRNA-based therapeutics. IFN-β exhibits direct anti-tumor functions, including inhibition of tumor-cell proliferation, induction of apoptosis, and suppression of angiogenesis [13,14,15]. Within the TME, IFN-β can promote the transition from an immunologically “cold” to a more inflamed “hot” state by enhancing dendritic cell maturation, lymph node migration, and cross-presentation of tumor antigens to CD8^+^ T cells [16,17,18,19]. It can also reduce the activity of immunosuppressive cell populations, including regulatory T cells and myeloid-derived suppressor cells, while promoting macrophage polarization toward a pro-inflammatory, anti-tumor M1-like phenotype [4,17,20].

Beyond its direct anti-tumor effects, IFN-β can also function as a potent vaccine adjuvant by shaping antigen-specific immune responses. IFN-β (type I interferon) signaling enhances the dendritic cell activation and antigen-presenting capacity, leading to more efficient priming of naïve CD8^+^ and CD4^+^ T cells [21,22]. IFN-β also promotes the survival and expansion of activated T cells and supports the differentiation of cytotoxic T lymphocytes capable of eliminating infected or malignant cells [23,24,25]. In addition, type I interferons contribute to the generation of robust humoral immunity by enhancing B-cell activation, germinal center formation, and antibody production [26]. Through these mechanisms, IFN-β can amplify both cellular and humoral immunity, making it an attractive candidate for incorporation into vaccine platforms designed to improve immunogenicity in cancer and infectious-disease settings [26,27].

Type I interferons have been evaluated clinically as immunomodulatory agents in both oncology and infectious diseases. IFN-α has been tested across multiple malignancies, primarily as a cancer vaccine adjuvant, as monotherapy in selected tumors, or in combination with chemotherapy [28,29,30,31,32,33,34,35,36]. A type I interferon-based gene therapy, nadofaragene firadenovec (Adstiladrin), is currently approved for BCG-unresponsive high-risk non-muscle-invasive bladder cancer [37]. Beyond oncology, type I interferons have also been explored as adjuvants for infectious-disease vaccines and have been shown to enhance antibody responses [38].

The temporal relationship between cytokine and antigen expression may be particularly important in mRNA-based vaccination. Type I IFN signaling can promote the activation of antigen-presenting cells [21,22], while simultaneously inducing an antiviral cellular state that may affect the stability and translation of exogenous mRNA [39,40]. These opposing effects suggest that the sequence of cytokine- and antigen-encoding mRNA delivery could influence the balance between antigen expression and immune activation. However, how the order of administration affects these responses remains insufficiently understood.

Here, we investigated whether nucleoside-modified mRNA-encoded *IFN-β* can function as a multifunctional immunomodulatory platform capable of inducing direct pro-apoptotic effects, reprogramming innate immune cells, and enhancing vaccine-associated immune activation. Using human cell lines and primary human immune-cell systems, we evaluated the capacity of *IFN-β* mRNA to induce interferon-stimulated responses, activate dendritic cells, repolarize macrophages, and exert timing-dependent effects on antigen expression and dendritic-cell activation in the context of mRNA vaccination.

## 2. Materials and Methods

### 2.1. IFN-β and OVA mRNA Production

A DNA sequence encoding human *IFN-β* was isolated from a healthy human DNA sample using following primers: forward, 5′-GTCACCATGGCTAGCAAGTGTCTC-3′; reverse, 5′-ACAGGCTAGGGCTAGCTCAGTTTCG-3′. The primers contained *NheI* restriction sites suitable for ligation into the expression vector, which contained the T7 polymerase promoter sequence and the desired UTRs. The full mRNA sequence is provided in the Supplementary Information (Figure S1). Correct cloning was determined by sequencing. In vitro transcription with co-transcriptional capping was performed using the NEB HiScribe High Yield RNA Kit (New England Biolabs, Frankfurt a. M., Germany) and the TriLink CleanCap AG 3’OMe analog (TriLink BioTechnologies, San Diego, CA, USA), with complete substitution of uridine by N1-methylpseudouridine. Following silica-membrane purification (NEB Monarch RNA Cleanup Kit) the RNA was enzymatically tailed using an E. coli poly(A) polymerase (NEB), resulting in a poly(A) tail longer than 100 bases. The mRNA subsequently was purified by silica-membrane purification and sorting with oligo d(T) magnetic beads (Dynabeads™ mRNA Purification Kit; Thermo Fisher Scientific, Waltham, MA, USA) to reduce dsRNA contamination.

### 2.2. Cultivation of HepG2 and HEK293T Cells

HepG2 cells were obtained from ATCC (HB-8065; Manassas, VA, USA) and cultured in 1× RPMI 1640 medium (Gibco, Grand Island, NY, USA) supplemented with 5 mM HEPES (Gibco), 2 mM L-glutamine (Gibco), 1% penicillin/streptomycin (Sigma-Aldrich, Steinheim am Albuch, Germany) and 10% FCS (Gibco). Cells were passaged once per week, with a medium change after 4 days of culture.

HEK293T cells were obtained from ATCC (CRL-3216) and cultured in DMEM (+4.5 g/L D-glucose, L-glutamine, without pyruvate; Gibco) supplemented with 10% FCS (Gibco), 1% sodium pyruvate (Sigma-Aldrich) and 1% penicillin/streptomycin (Sigma-Aldrich). Both cell lines were cultured in a humidified incubator at 37 °C and 5% CO_2_. Cells were passaged once per week to maintain them in culture.

HepG2 cells were selected as human hepatocellular carcinoma-derived model to investigate the pro-apoptotic effects of mRNA-encoded IFN-β in a tumor-derived cell line. HEK293T cells were included as a second human cell model because of their established suitability for transfection and recombinant protein expression, enabling assessment of the biological activity of the *IFN-β* mRNA construct in an additional cellular background. As HEK293T cells are immortalized and transformed, they were not considered a normal-cell control.

### 2.3. IP10 ELISA

Supernatants from transfected HepG2 and HEK293T cells were collected 24, 48, and 72 h after transfection and analyzed for secretion of the ISG CXCL10/IP10. The DuoSet ELISA Development System for human CXCL10/IP10 (R&D Systems, Minneapolis, MN, USA) was used according to the manufacturer’s protocol. CXCL10 concentrations were determined using a standard curve fitted with a four-parameter logistic model.

### 2.4. FITC Annexin V/Dead Cell Apoptosis Assay

To assess apoptosis induction in immortalized cell lines, the FITC Annexin V/Dead Cell Apoptosis Assay (Invitrogen, Carlsbad, CA, USA) was performed. Transfected HepG2 and HEK293T cells were harvested 24, 48, and 72 h after transfection and washed with DPBS. Annexin V and PI staining was performed according to the manufacturer’s protocol, followed by measurement with an LSR II flow cytometer (BD Biosciences, Heidelberg, Germany) and analysis using FlowJo software version 10.8. Apoptotic cells were quantified as Annexin V/PI double-positive cells.

### 2.5. Human Monocyte Isolation

Buffy coats from healthy donors were obtained from the blood bank (University Medical Center Mainz, Germany). Peripheral blood mononuclear cells (PBMCs) were isolated by density-gradient centrifugation using Histopaque-1077 (Sigma-Aldrich). Monocytes were isolated from the PBMCs using the human CD14 MicroBeads Kit (Miltenyi Biotec, Bergisch Gladbach, Germany) and cultured in 6-well plates in a humidified incubator at 37 °C and 5% CO_2_.

### 2.6. Human Monocyte-Derived Macrophage Differentiation

Monocytes isolated from human buffy coats were differentiated into monocyte-derived macrophages. Differentiation into M0-like macrophages was performed over 5 days in RPMI 1640 medium (Gibco) supplemented with 5 mM HEPES (Gibco), 2 mM L-glutamine (Gibco), 100 U/mL penicillin, 100 µg/mL streptomycin (Sigma-Aldrich), 10% FCS (Gibco), 10 ng/mL rhM-CSF (ImmunoTools, Friesoythe, Germany), and 1 ng/mL rhGM-CSF (ImmunoTools). Cells were seeded at 2 × 10^6^ cells per well in 6-well plates and cultured in a humidified incubator at 37 °C and 5% CO_2_. The medium was refreshed once after 2.5 days of incubation. For polarization into M2-like macrophages, cells were detached using a detachment buffer containing 0.5% BSA (Sigma-Aldrich), 5 mM EDTA (Sigma-Aldrich), and 4 mg/mL lidocaine hydrochloride monohydrate (Merck, Darmstadt, Germany), followed by incubation on ice for 30 min. Cells were seeded in 24-well plates at 215,000 cells per well. Polarization into M2-like macrophages was performed over 24 h using medium supplemented with 10 ng/mL rhIL-4 (ImmunoTools) and 10 ng/mL rhIL-13 (ImmunoTools).

### 2.7. Human Monocyte-Derived Dendritic Cells Differentiation

To generate MDDCs, monocytes isolated from buffy coats were cultured for 5 days in 1× RPMI 1640 medium (Gibco) supplemented with 10% FCS (Gibco), 2 mM L-glutamine (Gibco), 250 IU/mL rhIL-4 (ImmunoTools), and 800 IU/mL rhGM-CSF (ImmunoTools). Cells were seeded at 3 × 10^6^ cells per well in 6-well plates and cultured in a humidified incubator at 37 °C and 5% CO_2_. After 2.5 days, half of the medium was exchanged. The removed medium was collected in a tube and centrifuged at 400× *g* for 10 min at 4 °C. The old medium was discarded, and the cells were resuspended in fresh medium containing twice the concentration of cytokines before being transferred back to the original culture.

### 2.8. Transfection with Lipofectamine MessengerMAX

Cell lines, MDMs, and MDDCs were transfected 24 h after seeding or polarization. Cells were transfected with 500 ng mRNA combined with 1.5 µL Lipofectamine MessengerMAX (Thermo Fisher Scientific) according to the manufacturer’s instructions. Lipofectamine MessengerMAX alone (1.5 µL) was used as negative control to assess possible effects of the transfection reagent. *OVA* mRNA was used as a transfection control to distinguish IFN-β-specific effects from responses associated with mRNA transfection itself.

### 2.9. Western Blot Analysis

Total protein in cell lysates was quantified using the Qubit Protein Assay Kit (Invitrogen), and equal amounts of protein were loaded onto SDS-PAGE gel (Invitrogen) for size separation. Proteins were then transferred to a nitrocellulose membrane (Invitrogen) and blocked with 1× TBS containing 5% milk powder. Membranes were incubated overnight at 4 °C with anti-human IFN-β monoclonal antibody (BioLegend, San Diego, CA, USA; Cat. 514002) and anti-β-Tubulin antibody (Sigma-Aldrich, T7816) as a loading control. The following day, membranes were washed four times and incubated for 1 h at room temperature with anti-mouse IRDye 800 CW secondary antibody (LI-COR, Lincoln, NE, USA; 925-32210). After washing, membranes were scanned using the Empiria Studio^®^ Software 2.3 (LI-COR). The uncropped blots and corresponding intensity measurements are provided in the Supplementary Information (Figure S2).

### 2.10. Flow Cytometric Analysis

MDDCs were detached 24, 48, and 72 h after transfection using DPBS containing 2 mM EDTA and 0.5% BSA, followed by incubation on ice in the dark for 30 min. Cells were then collected and washed with FACS buffer. FC receptor blocking was performed using human IgG diluted in PBS before staining with fluorochrome-conjugated anti-human monoclonal antibodies against CD86 (BD Horizon™ V450), CD80 (PE), and HLA-DR (APC). After staining, cells were washed and further stained with the viability dye 7-AAD. Following an incubation for 5 min at room temperature in the dark, cells were acquired using an LSR II flow cytometer.

For co-transfection experiments, MDDCs were collected 48 h after transfection by transferring the supernatant into FACS tubes and detaching the adherent cells using detachment buffer (2 mM EDTA and 0.5% BSA) followed by incubation on ice in the dark for 30 min. Cells were then collected and washed with PBS. Viability staining was performed using the LIVE/DEAD™ Fixable Near-IR Dead Cell Stain Kit (Invitrogen), followed by incubation on ice for 30 min. After washing the cells with PBS, FC receptor blocking was performed using human IgG diluted in PBS before extracellular staining with fluorochrome-conjugated anti-human monoclonal antibodies against CD80 (BUV395), CD86 (V450) and HLA-DR (BV605). After incubation for 30 min at 4 °C, the samples were washed with PBS and permeabilized using the BD Cytofix/Cytoperm™ Fixation/Permeabilization Kit (BD Biosciences). Samples were then washed and stained intracellularly with antibodies against TNF-α (BUV737), IFN-γ (AF488), IL-12p70 (PE), IL-6 (PE-Cy7) and IL-8 (APC). After incubation for 30 min, samples were washed and acquired using a BD Symphony A3 cytometer.

Monocyte-derived macrophages were assessed 24 h, 72 h, and 6 days after transfection. Cells were detached using a detach buffer consisting of DPBS supplemented with 0.5% BSA, 5 mM EDTA, and 4 mg/mL Lidocaine hydrochloride monohydrate. For detachment, cells were incubated on ice for at least 30 min before being scraped from the surface and collected. Cells were subsequently washed with FACS buffer and FC receptor blocking was performed using human IgG. Staining was performed with fluorochrome-conjugated anti-human monoclonal antibodies against CD86 (BD Horizon™ V450), CD80 (PE), CD163 (PE-Cy™7), and CD206 (APC). As for MDDCs, cells were stained with 7-AAD after washing and then acquired by flow cytometry.

The complete list of antibodies is provided in the Supplementary Materials (Table S1).

Transfection efficiency was assessed by transfection with EGFP-encoding mRNA under the respective experimental transfection conditions and quantified by flow cytometry as the percentage of EGFP-positive cells. Transfection efficiencies for the individual cell types are provided in Supplementary Figure S3.

### 2.11. Cytokine and Chemokine Quantitation

Supernatants from transfected MDDCs and MDMs were collected 72 h after transfection, and cytokine and chemokine concentrations were measured. The LEGENDplex Human Essential Immune Response Panel (BioLegend) was used according to the manufacturer’s protocol. This panel includes 13 human cytokines/chemokines, including TNFα, IL-6 and free active TGF-β1. After sample acquisition on an LSR II flow cytometer (BD Biosciences), results were analyzed using the LEGENDplex Data Analysis Software Suite (BioLegend).

### 2.12. Statistical Analyses

All statistical analyses and graph generation were performed using GraphPad Prism version 11.0.0 (GraphPad Software, San Diego, CA, USA). Comparisons among three or more groups were performed using one-way ANOVA followed by Tukey’s multiple-comparison test or two-way ANOVA followed by Tukey’s multiple-comparison test, as appropriate. Data from in vitro experiments are presented as mean ± SD. Statistical significance was defined as follows: *, *p* < 0.05; **, *p* < 0.01; ***, *p* < 0.001; ****, *p* < 0.0001.

Unless otherwise stated, experiments were performed using independent biological replicates. For experiments with primary human cells, cells derived from individual donors were considered independent biological replicates. For experiments using cell lines, independently performed experiments were considered biological replicates. For CXCL10 measurements, technical replicate measurements were additionally performed within each biological replicate.

## 3. Results

### 3.1. IFN-β mRNA Induces Apoptosis and Interferon-Stimulated Responses in HEK293T and HepG2 Cells

**Figure 1:**
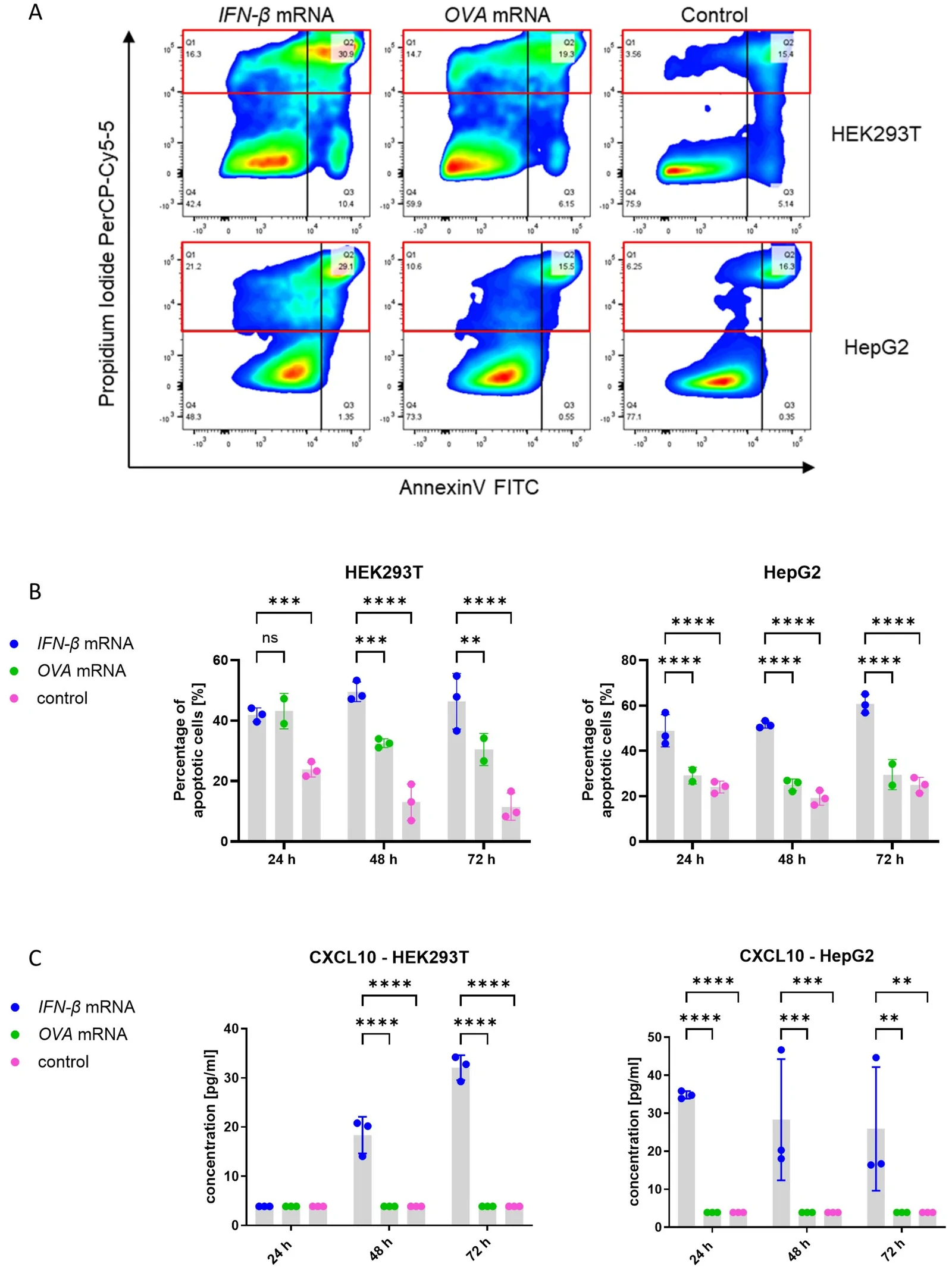
*IFN−β* mRNA induces apoptosis and CXCL10 secretion in HEK293T and HepG2 cells. (**A**) Representative flow-cytometry plots are shown using pseudocolor density, with blue through red indicating increasing event density. Cells positive for both PI and Annexin V (AnV), as well as PI-positive cells (indicated by the red box), were included in the analysis. Depicted here is data 48 h after transfection with the mRNA candidate as well as control mRNA. (**B**) Apoptosis in HEK293T and HepG2 cells was evaluated by Annexin V/PI Assay staining 24, 48, and 72 h after mRNA transfection. *OVA* mRNA-transfected cells served as an extraneous mRNA control. Statistical analysis was performed using a two-way ANOVA with multiple comparisons. The bar graphs show the mean ± SD from three independent experiments (*n* = 3). (**C**) CXCL10 secretion after mRNA transfection was analyzed by sandwich ELISA. Supernatants from HEK293T and HepG2 cells were collected 24, 48 and 72 h after transfection. Graph shows mean ± SD from three independent experiments measured in duplicate (*n* = 3). Significance was tested relative to the untreated control and *OVA* mRNA control groups: ns, not significant; **, *p* < 0.01; ***, *p* < 0.001; ****, *p* < 0.0001. (two-way ANOVA, Tukey’s multiple comparison test).

The ability of *IFN-β* mRNA to induce apoptosis was assessed by Annexin V (AnV) and propidium iodide (PI) staining in HEK293T and HepG2 cells. Compared with ovalbumin (OVA) mRNA-transfected controls, *IFN-β* mRNA increased the proportion of AnV^+^/PI^+^ cells, as reflected by the expanded upper right population in the flow cytometry plots. Flow cytometric analysis further showed a time-dependent decrease in viable AnV^−^/PI^−^ cells and a corresponding increase in AnV^+^/PI^+^ and PI^+^ populations after *IFN-β* mRNA transfection (Figure 1A).

In HepG2 cells, *IFN-β* mRNA transfection significantly increased the proportion of AnV^+^/PI^+^ and PI^+^ cells at 24, 48, and 72 h after transfection with the apoptotic fraction differing by up to approximately 30 percentage points compared with *OVA* mRNA. In HEK293T cells, a significant increase in apoptosis was observed at 48 and 72 h, but not at 24 h (Figure 1B). *OVA* mRNA transfection also increased apoptosis in HEK293T cells, although to a lesser extent than *IFN-β* mRNA, indicating that the IFN-β-specific effect is best interpreted relative to the *OVA* mRNA control.

#### IFN-β mRNA Stimulates CXCL10 Secretion in HEK293T and HepG2 Cells

To assess induction of interferon-stimulated responses, CXCL10 secretion was measured in HEK293T and HepG2 cells transfected with *IFN-β* mRNA or *OVA* mRNA, or left untreated.

In HEK293T cells, *IFN-β* mRNA significantly increased CXCL10 secretion at 48 and 72 h after transfection, with levels rising from below the detection limit to approximately 30 pg/mL in culture supernatants. In HepG2 cells, CXCL10 levels were significantly elevated at all three time points, reaching approximately 30 pg/mL, whereas CXCL10 remained below the detection limit following *OVA* mRNA transfection and in untreated controls (Figure 1C). These differences support an IFN-β-specific biological response to the expressed cytokine.

### 3.2. IFN-β mRNA Activates Monocyte-Derived Dendritic Cells

To investigate the immunomodulatory activity of *IFN-β* mRNA in primary human antigen-presenting cells, monocyte-derived dendritic cells (MDDCs) were used as a well-established human APC model. Given their central role in linking innate immune activation with antigen presentation and adaptive immune responses, MDDCs provide a relevant model system for assessing IFN-β-induced phenotypic activation.

#### 3.2.1. IFN-β Protein Production After mRNA Delivery

**Figure 2:**
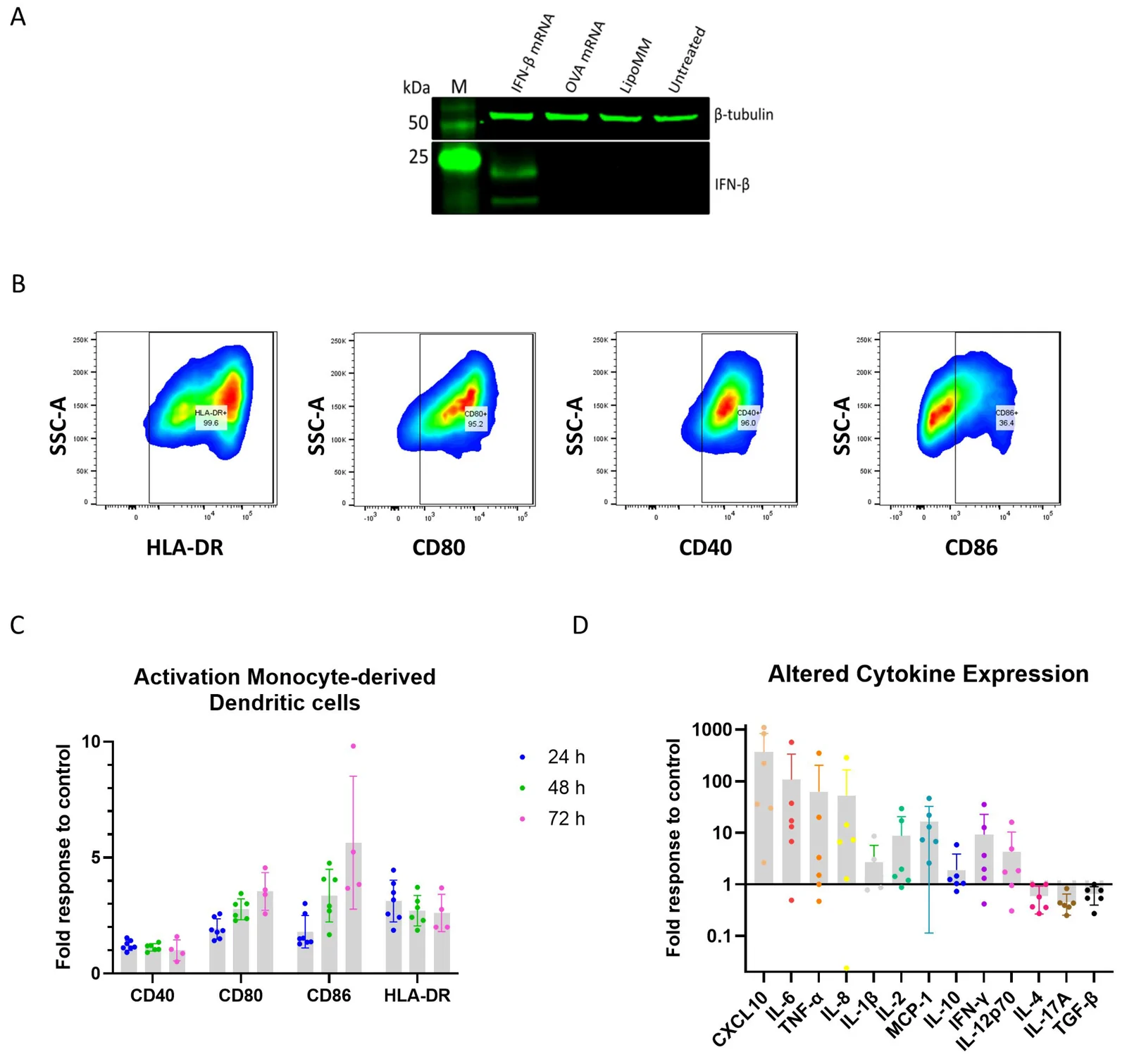
*IFN−β* mRNA activates monocyte-derived dendritic cells. (**A**) Western blot analysis of MDDC lysates collected 72 h after mRNA transfection. Equal amounts of protein (25 µg) were loaded and probed with anti-IFN-β and anti-β-tubulin antibodies. Uncropped Western blots and corresponding band intensity measurements are provided in Supplementary Figure S2. (**B**) Representative flow cytometry plots showing gating for activation marker expression on MDDCs, including HLA-DR, CD80, CD40, and CD86. Gates were defined using fluorescence-minus-one (FMO) controls, and marker expression was quantified as geometric mean fluorescence intensity (MFI). Pseudocolors indicate event density, ranging from blue (low density) to red (high density). Black boxes indicate the gates used to define marker-positive cell populations based on the corresponding FMO controls. The complete gating strategy is shown in the Supplementary Materials. (**C**) Monocytes were differentiated into MDDCs and transfected with mRNA. Activation marker expression was analyzed by flow cytometry 24, 48, and 72 h after transfection. Data represent mean ± SD from seven to eight healthy donors (N = 7/8), with each dot corresponding to one buffy coat. Values were normalized to the respective negative control. (**D**) Cytometric bead array analysis of cytokines in supernatants collected 72 h after MDDC transfection using the LEGENDplex™ platform (BioLegend). Data represent mean ± SD from six donors (N = 6), measured in duplicate and normalized to the corresponding negative control.

MDDCs, generated from peripheral blood of healthy donors, were transfected with *IFN-β* mRNA. Successful expression of the delivered mRNA was confirmed by Western blot analysis of MDDC lysates collected 72 h after transfection (Figure 2A). A distinct band corresponding to IFN-β was detected only in samples transfected with *IFN-β* mRNA, whereas no signal was observed in cells transfected with *OVA* mRNA or in other control conditions. Beta-tubulin served as a loading control and confirmed comparable protein input across samples.

#### 3.2.2. IFN-β mRNA Induces Activation of Monocyte-Derived Dendritic Cells

Expression of the MDDC surface markers HLA-DR, CD80, CD40 and CD86 was measured by flow cytometry 24, 48, and 72 h after transfection to assess the level of immune activation (Figure S4 and Figure 2B).

*IFN-β* mRNA-transfected MDDCs displayed increased expression of multiple activation markers compared with untreated controls. HLA-DR expression was elevated approximately three-fold at all analyzed time points, whereas CD80 and CD86 expression increased at 48 h and further increased at 72 h after transfection (Figure 2C). CD40 expression showed only minor changes relative to controls. These findings indicate enhanced surface expression of key DC activation markers after *IFN-β* mRNA transfection.

#### 3.2.3. Altered Cytokine Secretion Profile Following IFN-β mRNA Transfection

To evaluate functional changes in MDDC activation state, cytokine concentrations in cell culture supernatants collected 72 h after transfection were measured using a cytometric bead array (Figure 2D). *IFN-β* mRNA-transfected MDDCs showed markedly increased secretion of several pro-inflammatory mediators, including CXCL10, IL-6, TNF-α, IL-8, IL-1β, IL-2, MCP-1, IL-10, IFN-γ, and IL-12p70, compared with untreated controls. In contrast, secretion of IL-4, IL-17A, and TGF-β was reduced. Together, these findings indicate a cytokine profile consistent with enhanced pro-inflammatory activity in *IFN-β* mRNA-transfected MDDCs.

### 3.3. IFN-β mRNA Reprograms M2-like Macrophages Toward a Pro-Inflammatory Phenotype

#### 3.3.1. Selective IFN-β Protein Expression After IFN-β mRNA Transfection

**Figure 3:**
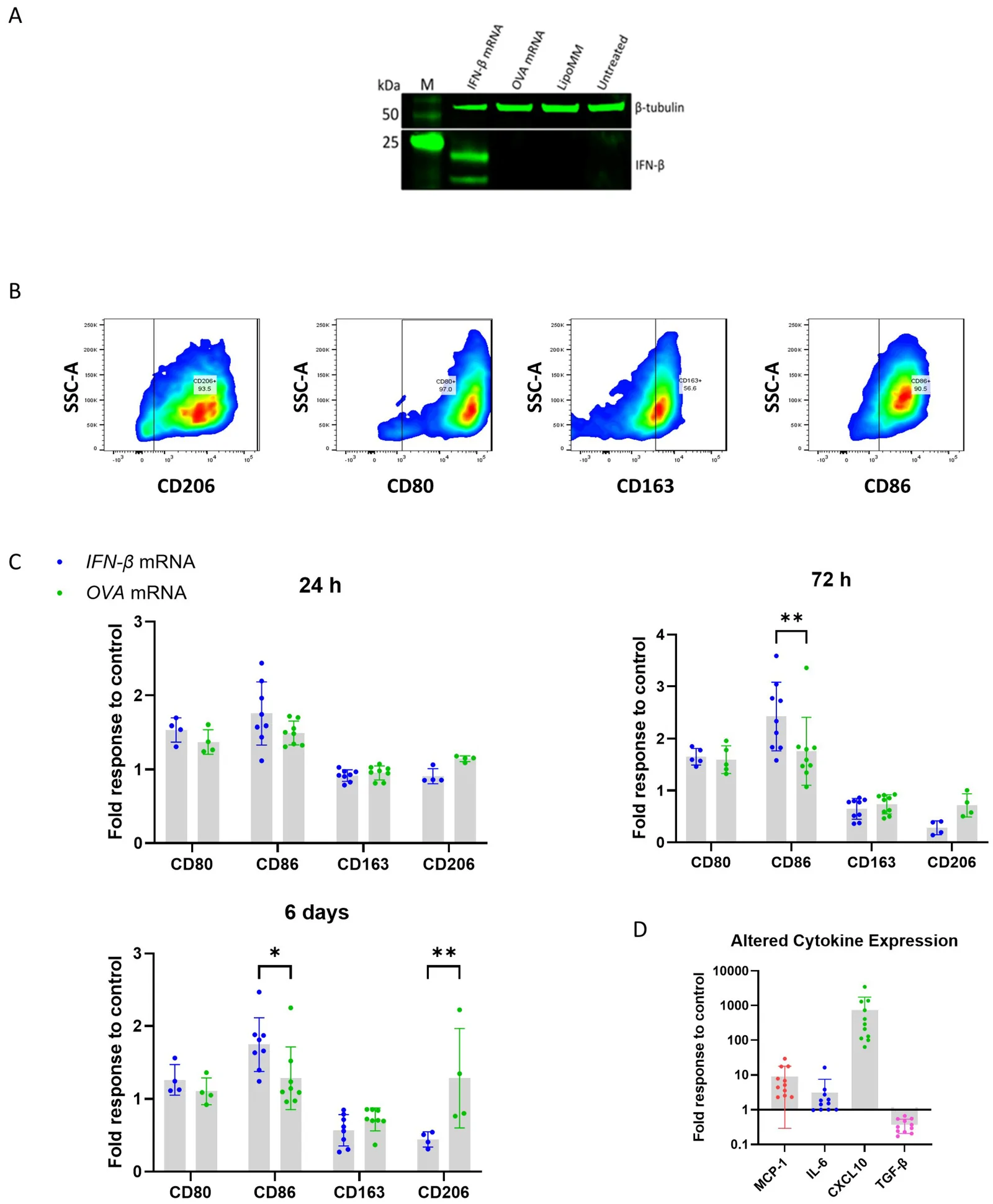
*IFN−β* mRNA repolarizes monocyte-derived macrophages. (**A**) Western blot analysis of MDM lysates collected 72 h after mRNA transfection. Equal amounts of protein (25 µg) were loaded, and β-tubulin served as the loading control. Uncropped Western blots and corresponding band intensity measurements are provided in Supplementary Figure S2. (**B**) Representative flow cytometry plots showing expression of macrophage polarization markers CD206, CD80, CD163, and CD86. Gates were defined using fluorescence-minus-one (FMO) controls. Pseudocolors indicate event density, ranging from blue (low density) to red (high density). Black boxes indicate the gates used to define marker-positive cell populations based on the corresponding FMO controls. The complete gating strategy is shown in the Supplementary Materials. (**C**) MDMs differentiated from PBMCs were polarized to an M2 phenotype and transfected with mRNA. Surface marker expression was analyzed by flow cytometry at 24 h, 72 h, and 6 days after transfection. Data represent mean ± SD from either four donors (CD80, CD206; N = 4) or eight donors (CD163, CD86; N = 8), with each dot representing one buffy coat normalized to its untreated control. Statistical significance was determined relative to *OVA* mRNA-transfected cells (* *p* < 0.05; ** *p* < 0.01; two-way ANOVA with Tukey’s multiple comparisons test). (**D**) Cytometric bead array analysis of cytokines in supernatants collected 72 h after MDM transfection using the LEGENDplex™ platform (BioLegend). Data represent mean ± SD from 11 donors (N = 11), measured in duplicate and normalized to untreated controls.

To confirm successful delivery and translation of *IFN-β* mRNA, protein expression was analyzed by Western blot in lysates from M2-polarized monocyte-derived macrophages (MDMs) collected 72 h after transfection. A distinct signal corresponding to the IFN-β molecular weight was detected only in cells transfected with *IFN-β* mRNA, whereas no signal was observed in cells transfected with *OVA* mRNA, treated with Lipofectamine MessengerMAX alone, or left untreated (Figure 3A). These results demonstrate efficient intracellular production of IFN-β protein in MDMs after mRNA delivery.

#### 3.3.2. IFN-β mRNA Shifts Surface Marker Profile of M2-like Macrophages

Changes in macrophage phenotype were evaluated by flow cytometric analysis of surface markers associated with M1-like (CD80, CD86) and M2-like (CD163, CD206) states at 1 day, 3 days and 6 days after transfection (Figure S5 and Figure 3B). *IFN-β* mRNA-transfected MDMs showed increased expression of the M1-associated marker CD80 when compared with *OVA* mRNA controls, although this increase did not reach statistical significance. In contrast, CD86 expression was significantly elevated 72 h and 6 days after *IFN-β* mRNA transfection (Figure 3C). By contrast, M2-associated markers decreased over time. CD163 expression was reduced at day 6 after *IFN-β* mRNA transfection, although not significantly, whereas CD206 expression was significantly decreased at day 6 compared with *OVA* mRNA controls and showed a similar trend at 72 h (Figure 3C). Together, these data indicate a shift in macrophage surface marker expression toward a pro-inflammatory phenotype after *IFN-β* mRNA treatment.

#### 3.3.3. IFN-β mRNA Induces a Pro-Inflammatory Secretory Profile

To assess functional changes in macrophage activity, cytokine and chemokine secretion was measured in cell culture supernatants collected 72 h after transfection using a cytometric bead array (Figure 3D). *IFN-β* mRNA-transfected MDMs showed increased secretion of the pro-inflammatory mediators CXCL10, MCP-1, IL-6, and IL-2 compared with control conditions. In contrast, levels of the immunoregulatory cytokine TGF-β were reduced. No substantial differences were detected for the remaining analytes included in the panel. These findings demonstrate that *IFN-β* mRNA transfection is associated with broad changes in the secretory profile of M2-polarized macrophages.

### 3.4. Timing-Dependent Adjuvant Activity of IFN-β mRNA in Human MDDCs

To determine whether timing of IFN-β delivery relative to antigen-encoding mRNA influences MDDC activation and antigen expression, we compared sequential transfection regimens differing in the order of administration. A 24 h interval was chosen as a predefined proof-of-concept condition to clearly separate the two transfection events and allow assessment of administration order. Human MDDCs were transfected with two distinct mRNA components: an adjuvant-position mRNA and an antigen-position mRNA. In the adjuvant position, *IFN-β* was compared with *OVA* mRNA, which served as a non-adjuvant control mRNA to account for non-specific effects of mRNA transfection and translation. In the antigen position, *EGFP* mRNA was used as a reporter protein to quantify mRNA translation, whereas *OVA* mRNA served as control for conditions in which fluorescent antigen tracking was not required.

**Figure 4:**
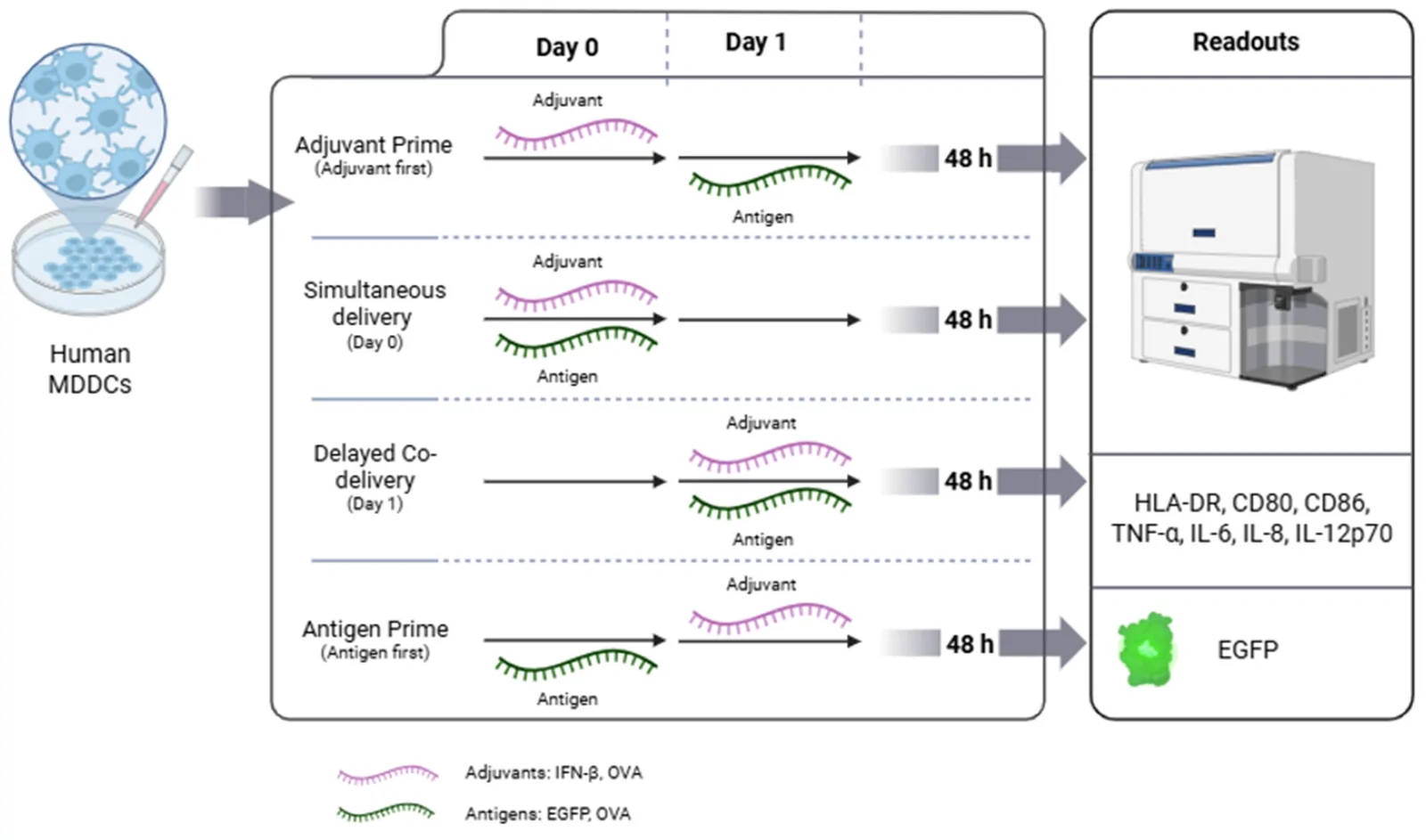
Experimental design used to evaluate the adjuvant activity of *IFN-β* mRNA in human monocyte-derived dendritic cells (MDDCs). Cells were transfected with adjuvant mRNA (*IFN-β* or *OVA* control) and antigen-encoding mRNA (*EGFP* or *OVA*) under different temporal regimens, including adjuvant priming before antigen delivery, simultaneous delivery, delayed co-delivery, and antigen-first delivery followed by adjuvant mRNA (Antigen Prime). Forty-eight hours after the second transfection, MDDC activation and antigen expression were assessed by flow cytometry. Created in BioRender. Fraude-El Ghazi, S. (2026) https://BioRender.com/x46p9cv.

The two mRNA components were administered according to four defined temporal regimens, as depicted in Figure 4. In the Adjuvant Prime regimen, MDDCs first received the adjuvant-position mRNA, either *IFN-β* mRNA or *OVA* control mRNA, followed by antigen-encoding mRNA after 24 h. This condition tested whether prior *IFN-β* mRNA exposure enhances immune activation but affects subsequent antigen expression.

In the Simultaneous Delivery regimen, the adjuvant-position mRNA and antigen-encoding mRNA were delivered together at the first transfection time point. This condition tested whether *IFN-β* mRNA can provide adjuvant activity when present at the same time as antigen expression.

In the Delayed Co-delivery regimen, both mRNA components were again delivered together, but at the later transfection time point. This condition controlled for the effect of delayed transfection while maintaining simultaneous exposure to adjuvant-position and antigen-position mRNAs.

In the Antigen Prime regimen, MDDCs first received antigen-encoding mRNA, followed by adjuvant-position mRNA on the next day. This condition tested whether antigen expression established before *IFN-β* mRNA exposure would be preserved despite subsequent IFN-β-mediated immune activation.

Thus, *OVA* mRNA served two experimental control functions depending on its position in the regimen: as an irrelevant antigen/protein control in the antigen position, and as non-adjuvant protein control. For all regimens, cells were analyzed 48 h after the second transfection.

#### 3.4.1. Robust Activation of MDDCs Following IFN-β mRNA Delivery

**Figure 5:**
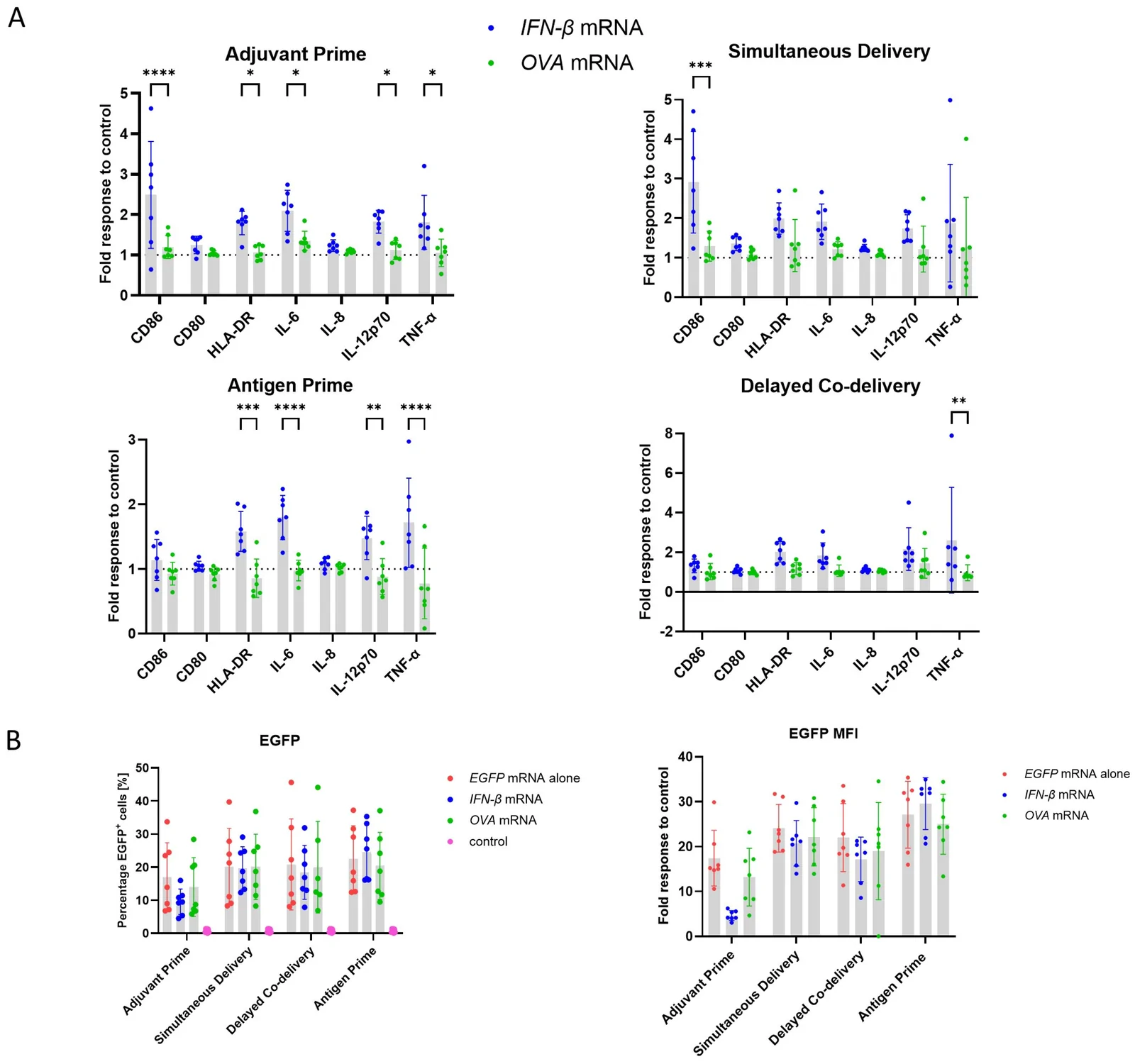
Adjuvant effects of *IFN−β* mRNA on MDDC activation and antigen expression. (**A**) Monocytes were differentiated into MDDCs and transfected with adjuvant mRNA and antigen mRNA (*OVA*) according to the experimental scheme in Figure 4. Activation marker expression was analyzed by flow cytometry 48 h after the second transfection. Data represent mean ± SD from seven independent donors (N = 7). Values were normalized to the respective untreated control for each donor. Statistical significance was determined relative to *OVA* controls (* *p* < 0.05; ** *p* < 0.01; *** *p* < 0.001; **** *p* < 0.0001; two-way ANOVA). (**B**) MDDCs were transfected with *EGFP* antigen mRNA together with *IFN-β* adjuvant mRNA or *OVA* control mRNA according to the experimental scheme. *EGFP* translation efficiency was assessed by flow cytometry 48 h after transfection. Data represent mean ± SD from seven donors (N = 7). Mean fluorescence intensity (MFI) values were normalized to the corresponding negative control for each donor.

Expression of the activation markers CD86, CD80, and HLA-DR was assessed by flow cytometry (Figure S6). *IFN-β* mRNA increased expression of all three markers in the Adjuvant Prime and Simultaneous Delivery settings compared with *OVA* controls. CD86 expression was significantly elevated in both conditions, whereas HLA-DR showed a significant increase primarily in the Adjuvant Prime setting. HLA-DR was also significantly upregulated when *IFN-β* mRNA was administered on the second day. Changes in CD80 expression did not reach statistical significance (Figure 5A).

#### 3.4.2. Enhanced Intracellular Pro-Inflammatory Cytokine Production

Intracellular staining revealed increased production of pro-inflammatory cytokines after *IFN-β* mRNA treatment. IL-6, IL-12p70 and TNF-α were significantly elevated in both the Adjuvant Prime and Antigen Prime conditions. TNF-α expression was also significantly increased in the Delayed Co-delivery setting. IL-8 showed less pronounced changes across conditions (Figure 5A).

#### 3.4.3. Time-Dependent Effects on Antigen Expression

To assess whether delivery of *IFN-β* mRNA has an impact on antigen translation, *EGFP* mRNA was used as a reporter protein under the same timing regimens (Figure S7 and Figure 5B). Priming MDDCs with *IFN-β* mRNA one day before antigen delivery (Adjuvant Prime) reduced EGFP expression, as reflected by both a lower proportion of EGFP-positive cells and decreased mean fluorescence intensity compared with control conditions. In contrast, the other three regimens showed minimal reduction in EGFP expression. In the Antigen Prime condition, EGFP levels were modestly higher than in controls (Figure 5B).

Together, these results demonstrate that *IFN-β* mRNA exerts potent adjuvant effects on human MDDCs in a strongly timing-dependent manner, with timing determining both immune activation and preservation of antigen expression. Across MDDC and MDM transfection experiments, viability remained stable, indicating that the mRNA constructs did not decrease cell viability (Figure S8).

## 4. Discussion

This study characterizes the in vitro activity of nucleoside-modified, human IFN-β-encoding mRNA across tumor-derived cell lines and primary human immune cells and identifies a delivery-timing principle that is directly relevant to the co-formulation of cytokine-encoding and antigen-encoding mRNAs. By integrating readouts of apoptosis, ISG induction, dendritic cell activation, macrophage polarization, and antigen expression under defined co-transfection regimens, our work provides functional and pharmaceutically relevant characterization of *IFN-β* mRNA as an immunomodulatory component for mRNA-based vaccination strategies. The construct was produced as N1-methylpseudouridine-substituted mRNA capped with CleanCap AG (3′OMe) to minimize construct-intrinsic innate sensing and to isolate the biological activity of the translated IFN-β protein from generic mRNA immunogenicity [28,29,41].

We first assessed whether mRNA-encoded *IFN-β* retains canonical pro-apoptotic activity. *IFN-β* mRNA induced significant apoptosis and necrosis in HepG2 cells and a more modest, time-dependent effect in HEK293T cells, consistent with the well-described ability of type I interferons to engage pro-apoptotic ISGs, including TRAIL, XAF1, and members of the OAS/RNase L axis [42]. Recombinant IFN-β protein has been reported to induce TRAIL expression and reduce viability in HepG2 [43,44], and similar effects have been documented in breast cancer (MDA-MB-231, HS578T) and multiple myeloma cell lines [45,46], although in each case using protein rather than mRNA delivery. Kimura et al. more recently reported an mRNA-based strategy using murine *Ifnb1* mRNA in lung- and oral-carcinoma cell lines, with statistically significant apoptosis in oral-carcinoma cells only [47]. Our findings extend this literature by showing that human IFN-β expressed from a modified-nucleoside mRNA construct is pro-apoptotic activity in the investigated cell models, demonstrating that biologically active IFN-β can be generated following mRNA transfection.

Two features of these data warrant explicit acknowledgement. First, in HEK293T, *OVA* control mRNA also produced a measurable increase in AnV/PI-positive cells, so the IFN-β-specific effect is best interpreted from the delta over the OVA control rather than from raw values. Such IFN-β independent effects may reflect cellular responses associated with mRNA transfection and/or the transfection reagent itself. This is consistent with the well-documented ability of Lipofectamine-mediated mRNA transfection to activate cytosolic RNA sensors and to trigger stress-response pathways independently of the encoded protein [39,41]. Second, the apoptotic response in HEK293T reached significance only at 48–72 h. Together, these observations frame *IFN-β* mRNA as a genuine but context-dependent inducer of tumor-cell death that will require validation with clinically relevant delivery vehicles.

A relevant feature of mRNA-based IFN-β expression is that the protein is synthesized and modified by the host cell’s own machinery. IFN-β carries a single N-linked glycan at Asn-80 that contributes to biological activity, solubility, secretion, and stability; deglycosylation reduces specific activity by roughly one order of magnitude in cell-based assays [48]. However, because recombinant IFN-β was not included as a matched active comparator in the present study, these considerations should be regarded as potential advantages of the mRNA platform rather than experimentally demonstrated benefits. Accordingly, no conclusion can be drawn regarding the relative efficacy or superiority of mRNA-encoded IFN-β over recombinant protein administration.

In addition to its direct pro-apoptotic activity, IFN-β orchestrates immune responses through the induction of ISGs and chemokines. CXCL10 (IP10) is a type I IFN-inducible chemokine that guides migration of T helper 1 cells and recruits natural killer (NK) cells [49]. In the TME, signaling through the CXCL10-CXCR3 axis helps shape an inflammatory milieu by attracting multiple immune-cell subsets to lesions [50]. IFN-β induces CXCL10 expression through the activation of the type I interferon receptor (IFNAR), which triggers the JAK1/TYK2-STAT1/STAT2 signaling cascade and formation of the ISGF3 transcriptional complex, ultimately driving the transcription of ISGs including CXCL10 [42,49]. Consistent with this pathway, *IFN-β* mRNA-transfected HepG2 cells, HEK293T cells, MDMs, and MDDCs produced detectable CXCL10, confirming that the translated cytokine was biologically active and capable of inducing downstream ISG responses.

IFN-β also plays a central role in shaping the antigen-presenting cells’ activity. It promotes monocyte differentiation into dendritic cells and upregulates major histocompatibility complex (MHC) molecules together with co-stimulatory receptors [51,52]. To test whether our mRNA-encoded *IFN-β* retains this functionality in human immune cells, we transfected MDDCs immediately after differentiation. Human MDDCs are a well-established in vitro model of antigen-presenting cells and are particularly relevant for evaluating mRNA-based immunotherapies because dendritic cells mediate antigen presentation and T-cell priming after vaccination [53,54]. *IFN-β* mRNA enhanced expression of key activation markers and increased secretion of pro-inflammatory cytokines, demonstrating functional activation of human MDDCs. In the context of vaccination, activation of dendritic cells is critical for efficient antigen presentation and co-stimulatory signaling, thereby promoting robust priming of antigen-specific T cell responses and enhancing overall vaccine immunogenicity [55]. These findings align with those of Huang et al., who reported upregulation of CD80, CD86, and HLA-DR after recombinant IFN-β treatment. In contrast to their observation of Th2 skewing, our data suggest a more Th1-associated inflammatory profile, supported by reduced IL-4 secretion [56].

Macrophages represent another key innate immune population that is strongly influenced by interferon signaling. The TME is a multifaceted, immunosuppressive niche in which several stromal and immune populations collaborate to shield cancer cells from immune attack [1,2,3,4,5,6]. Macrophages constitute the major immune-cell subset in this milieu—nearly 50% of all TME cells—and are commonly categorized along a functional spectrum that includes classically activated, pro-inflammatory M1-like states and alternatively activated, immunoregulatory M2-like states [57,58]. Because M2-like macrophages can dampen anti-tumor immunity and support tumor growth, repolarizing them toward an M1-like phenotype is an attractive therapeutic strategy [59].

Following transfection of M2-polarized human macrophages with *IFN-β* mRNA, we observed coordinated changes in surface markers and cytokine secretion consistent with a shift toward a pro-inflammatory phenotype. These patterns are in line with established macrophage marker profiles [60,61]. Recombinant IFN-β has previously been shown to repolarize murine bone-marrow-derived macrophages [62] and tumor-associated macrophages in an LLC-OVA mouse model [47]. Our study extends these findings by demonstrating that mRNA-encoded IFN-β can induce similar shifting toward a pro-inflammatory phenotype in primary human macrophages, supporting its translational relevance.

One hallmark of this phenotypic shift was reduced production of the immunoregulatory TGF-β in both MDMs and MDDCs. TGF-β maintains immune homeostasis by suppressing inflammatory responses and promoting immune tolerance; it supports regulatory T-cell differentiation and is commonly associated with immunosuppressive environments, including M2-like macrophage polarization and tumor-mediated immune evasion [63]. Although TGF-β is not directly regulated by IFN-β, type I interferon signaling can antagonize TGF-β/SMAD signaling through STAT1-dependent inhibition of SMAD transcriptional activity and induction of inhibitory regulators such as SMAD7, thereby limiting TGF-β-mediated immunosuppressive responses [63,64,65]. In dendritic cells, TGF-β production is typically associated with tolerogenic states, whereas inflammatory stimuli and type I interferons tend to suppress tolerogenic programming and promote immunogenic activation [63,66]. Thus, the reduced TGF-β secretion observed after *IFN-β* mRNA treatment is consistent with a loss of tolerogenic immune programming and supports the conclusion that IFN-β promotes a more pro-inflammatory, immunostimulatory phenotype in both dendritic cells and macrophages.

These immunostimulatory effects are particularly relevant for vaccine formulation, where activation of innate immune pathways is essential for the induction of robust adaptive immunity. Effective vaccination relies on judicious adjuvantation, but not every immunostimulant is compatible with the mRNA platform: some adjuvants can impair mRNA translation and thereby reduce antigen expression [39,67,68]. Type I interferons are well-established modulators of vaccine responses because they can enhance dendritic cell activation and promote antigen-specific T-cell priming, and recent work has shown that locally produced IFN-β at the injection site helps shape the early innate immune activation and adaptive immune responses after mRNA vaccination [69]. Despite this interest, the immunological consequences of *IFN-β* mRNA delivery in primary human immune cells remain incompletely defined.

Our co-transfection experiments in human MDDCs demonstrate that *IFN-β* mRNA acts as a potent innate immune activator whose impact on antigen expression depends strongly on the sequence of delivery. Antigen expression, measured here as EGFP fluorescence, was preserved when the antigen-encoding mRNA preceded IFN-β administration, whereas prior IFN-β exposure reduced the translational efficiency of a subsequently delivered mRNA. The temporal experiments were restricted to a pre-defined 24 h interval and were designed to assess the effect of administration order rather than to identify an optimal interval between IFN-β and antigen-encoding mRNA delivery. Because mRNA translation, IFN-β production, and downstream interferon-stimulated responses may follow distinct kinetics, systematic evaluation of shorter and longer intervals will be required to define the optimal temporal window. In addition, IFN-β production and downstream signaling kinetics were not directly quantified for the individual temporal regimens. Thus, although MDDC activation differed depending on the sequence of administration, these differences cannot be directly correlated with the concentration or duration of IFN-β exposure. A potential explanation for this timing-dependent effect is the antiviral state induced by type I IFN signaling. IFN-β-induced ISG have been reported to restrict translation of incoming cytoplasmic mRNA through pathways involving PKR/eIF2α, IFIT proteins, and 4E-BP1 while additional mechanisms such as RNase L- and ZAP-mediated RNA degradation may also contribute [39,40,70]. These mechanisms were not directly investigated in the present study and therefore remain hypotheses requiring further validation. Because our EGFP readout reflects the composite of translation, folding, maturation, and protein stability, we cannot distinguish between translational suppression and accelerated mRNA turnover based on fluorescence data alone; targeted qRT-PCR of EGFP mRNA at matched timepoints and orthogonal antigen-presentation readouts (for example, MHC-I:SIINFEKL detection with the 25-D1.16 antibody in appropriate H-2K^b^-expressing systems) will resolve this in follow-up work. Notwithstanding this, the observation that the sequence of cytokine mRNA delivery relative to antigen mRNA influenced whether antigen expression was preserved or reduced under the investigated conditions highlights the importance of administration sequence when designing combination mRNA formulations.

In addition to its immunological activity, mRNA delivery offers practical advantages over conventional protein-based cytokine therapy, particularly with respect to safety and tolerability. Systemic administration of recombinant IFN-β frequently causes flu-like symptoms, asthenia, hypersensitivity, elevated liver enzymes, and depression [71]. Protein therapy can also be limited by neutralizing antibodies, a problem well documented in patients with multiple sclerosis receiving IFN-β [72,73,74]. Proteins synthesized in situ from mRNA undergo native post-translational processing and may be less likely to be perceived as foreign, supporting the potential of mRNA therapeutics for repeated dosing [75,76,77].

From a pharmaceutical-development perspective, our findings position *IFN-β* mRNA as a candidate active pharmaceutical ingredient (API) that must next be paired with a clinically translatable delivery vehicle. In the present study, mRNA was delivered using Lipofectamine MessengerMAX to isolate the biological activity of the encoded IFN-β from formulation variables; clinically relevant delivery will require lipid nanoparticle (LNP) formulations with defined size, polydispersity, encapsulation efficiency, and organ-tropism profiles. This is particularly relevant for IFN-β because the broad expression of type I interferon receptor may result in off-target effects following systemic cytokine exposure. Local or intra-tumoral delivery of *IFN-β* mRNA may therefore provide a strategy to concentrate cytokine expression within the tumor microenvironment while limiting systemic exposure. Targeted LNP formulations could further improve spatial control by directing mRNA expression toward selected tissues or immune-cell populations, thereby potentially increasing the therapeutic window. Accordingly, future studies should evaluate the present *IFN-β* mRNA construct using clinically relevant and, where feasible, targeted LNP formulations to determine how delivery characteristics influence biodistribution, cell-type specificity, and biological activity in vivo. LNP composition and PEG-lipid content can be engineered for preferential delivery to hepatocytes, splenic myeloid cells, or draining-lymph-node antigen-presenting cells [70], each of which is a plausible target compartment for the different indications the platform could serve, such as hepatic tumors and chronic viral infection, cancer immunotherapy in the periphery, and injection-site adjuvantation of prophylactic mRNA vaccines.

In addition to spatial control, the timing and duration of IFN-I signaling are important determinants of its biological effects. Acute and appropriately timed IFN-I responses can support antiviral and antitumor immunity, whereas prolonged, excessive, delayed, or dysregulated signaling may contribute to pathological inflammation, immune dysfunction, and treatment resistance [78,79]. This context-dependent duality has been described in both viral infection, including COVID-19, and cancer and emphasizes that therapeutic IFN-β exposure must be controlled temporally as well as spatially. The timing-dependent effects observed in the present study, in which the sequence of IFN-β and antigen-encoding mRNA administration influenced antigen expression and MDDC activation, further support this consideration.

The construct characterized here therefore represents an in vitro proof of activity that should next be evaluated using clinically relevant and, where appropriate, targeted LNP delivery, together with biodistribution profiling and dose-timing pharmacokinetic studies.

## 5. Limitations and Future Work

Although this study combines extensive in vitro analyses, additional investigations in diverse disease-relevant in vivo systems will be necessary to fully establish the translational potential of *IFN-β* mRNA. Monocyte-derived cells exhibit considerable donor-to-donor variability; although samples from multiple healthy donors were included to strengthen the robustness of our findings, larger donor cohorts and analyses using patient-derived immune cells will be important to better capture clinically relevant immune heterogeneity. A further limitation is the absence of a direct comparison with recombinant IFN-β under matched bioactivity conditions, which precludes conclusions regarding potential functional advantages of mRNA-encoded over protein-based IFN-β delivery. Furthermore, our in vitro experiments revealed timing-dependent effects of *IFN-β* mRNA on dendritic cell activation and antigen expression in MDDCs, suggesting that the timing of cytokine mRNA exposure relative to antigen delivery may critically influence downstream immune responses.

However, the temporal experiments were restricted to a predefined 24 h interval, and IFN-β protein levels and downstream signaling kinetics were not quantified for the individual temporal regimens. Therefore, the observed differences in MDDC activation cannot be directly correlated with the magnitude or duration of IFN-β exposure. Future studies incorporating different administration intervals and time-resolved measurements of IFN-β production and downstream interferon signaling will be required to define the optimal temporal window and establish this relationship.

## 6. Conclusions

Our data show that nucleoside-modified mRNA encoding human IFN-β is biologically active in the investigated human cell models, inducing time-dependent apoptosis and CXCL10 secretion in HepG2 and HEK293T cells, promoting MDDC activation, shifting M2-like macrophages toward a pro-inflammatory phenotype, and exert timing-dependent effects on antigen expression and MDDC activation. The observation that the sequence of cytokine- and antigen-encoding mRNA influenced antigen expression under the investigated conditions highlights temporal sequence as an important parameter for the design of combination mRNA formulations. The work presented here is an in vitro proof of activity for a candidate cytokine-mRNA active pharmaceutical ingredient; formulation-focused studies with clinically relevant LNP delivery, biodistribution and pharmacokinetic profiling, patient-derived immune cells, and in vivo efficacy models are required before translational potential can be established. Nonetheless, our findings support the further development of cytokine-encoding mRNA as a programmable tool for immune modulation in cancer immunotherapy and mRNA vaccine formulation.

## Data Availability

Data supporting the findings of this study are available from the corresponding author, M.L.C., upon request.

## Supplementary Materials

The following supporting information can be downloaded at: https://www.mdpi.com/article/10.3390/vaccines14090829/s1, Figure S1: In silico-designed DNA sequence of the human *IFN-β* gene (M28662) flanked by 5′ and 3′ hemoglobin UTRs, Figure S2: Uncropped Western blot and signal intensity analysis of IFN-β expression in MDMs and MDDCs, Table S1: Inventory of antibodies used for flow cytometry analysis, Figure S3: Transfection efficiency of the investigated cell types, Figure S4: Gating strategy for MDDC activation, Figure S5: Gating strategy for MDM repolarization, Figure S6: Gating strategy for surface and intracellular staining in co-transfected MDDCs, Figure S7: Gating Strategy for EGFP reporter mRNA expression in co-transfected MDDCs, Figure S8: Assessment of viability in MDDCs as well as MDMs after mRNA transfection.
