# Supplementary material for: Timing-Dependent Immunostimulatory Activity of Human IFN-β mRNA in Primary Human Myeloid Cells
Source: Vaccines (Basel). 2026 Sep 20;14(9):829. doi: 10.3390/vaccines14090829 (PMC13611373; doi:10.3390/vaccines14090829)
Supplement: Supplementary file 1 [file vaccines-14-00829-s001.zip › vaccines-4534293-supplementary.pdf]

Supplementary Information

Timing-dependent genetic adjuvant activity of human *IFN-β* mRNA in primary human myeloid cells

Silvia Fraude-El Ghazi, Maria José Limeres, Rocio Gambaro, Ana Pena Vaquero, Dirk Prawitt, German Islan, Stephan Gehring and Maximiliano L. Cacicedo *

5'-Hbb-UTR

ACCACCAAGCTTACATTTGCTTCTGACACAACTGTGTTCACTAGCAACCTCAAACAGCCACCATGGCTAGCAAGTGTCTCCTCCA
AATTGCTCTCCTGTTGTGCTTCTCCACTACAGCTCTTTCCATGAGCTACAACTTGCTTGGATTCCTACAAAGAAGCAGCAATTTTC
AGTGTCAGAAGCTCCTGTGGCAATTGAATGGGAGGCTTGAATACTGCCTCAAGGACAGGATGAACTTTGACATCCCTGAGGAG
ATTAAGCAGCTGCAGCAGTTCCAGAAGGAGGACGCCGCATTGACCATCTATGAGATGCTCCAGAACATCTTTGCTATTTTCAGA
CAAGATTCATCTAGCACTGGCTGGAATGAGACTATTGTTGAGAACCTCCTGGCTAATGTCTATCATCAGATAAACCATCTGAAGA
CAGTCCTGGAAGAAAAACTGGAGAAAGAAGATTTCACCAGGGGAAAACTCATGAGCAGTCTGCACCTGAAAAGATATTATGG
GAGGATTCTGCATTACCTGAAGGCCAAGGAGTACAGTCACTGTGCCTGGACCATAGTCAGAGTGGAAATCCTAAGGAACTTTT
ACTTCATTAACAGACTTACAGGTTACCTCCGAAACTGAGCTAGCTTTCTTGCTGTCCAATTTCTATTTAAAGGTTCCTTTGTTCCC
TAAGTCCAACTACTAAACTGGGGGATATTATGAAGGGCCTTGAGCATCTGGATTCTGCCTAATAAAAAACATTATTTTCATTGC
AAGAATTCTCTAGAACCACC

3'-Hbb-UTR

Figure S1: In silico-designed DNA sequence of the human *IFN-β* gene (M28662) flanked by 5' and 3' hemoglobin UTRs. The open reading frame is shown in blue, *Nhe*I restriction sites are shown in green, and the start and stop codons are indicated in red.

A

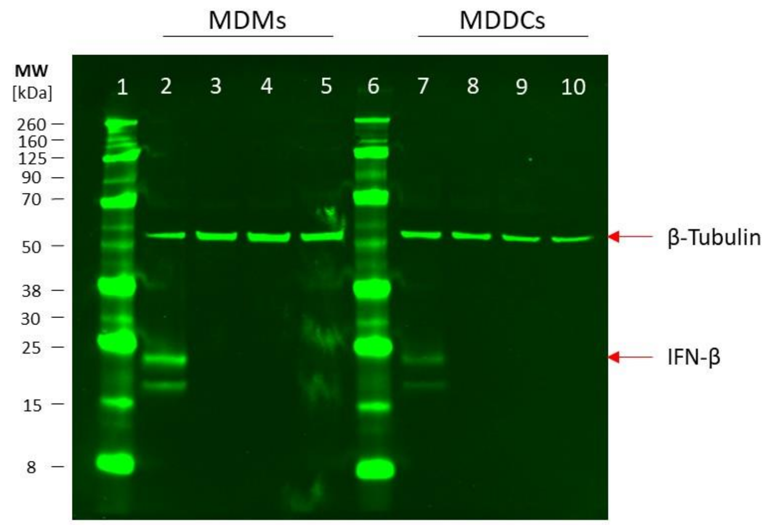

B

			IFN-β		β-Tubulin	
Cells	Lane	Name	MW	Signal	MW	Signal
MDMs	2	IFN-β	22,1401803	1586,23418	59,4664804	666,36924
	3	OVA	24,9011218	-4,37252832	57,6589685	1754,39166
	4	LipoMM	24,8586388	-12,3657125	55,9231314	2059,28746
	5	Control	25,0459776	183,820557	57,0803336	1899,50781
MDDCs	7	IFN-β	22,9190356	443,389809	59,8555412	1078,70976
	8	OVA	23,1085508	-11,7545457	56,8123077	983,214263
	9	LipoMM	24,3277375	-4,74792463	54,0578779	667,433526
	10	Control	18,4378547	-6,91528826	54,0578779	491,366232

Figure S2: Uncropped Western blot and signal intensity analysis of IFN-β expression in MDMs and MDDCs. A) Uncropped Western blot showing IFN-β and β-Tubulin expression in monocyte-derived macrophages (MDMs) and monocyte-derived dendritic cells (MDDCs) after transfection with IFN-β mRNA, OVA control mRNA, Lipofectamine MessengerMAX alone, or no treatment. IFN-β was detected only in IFN-β mRNA-transfected cells, whereas β-Tubulin served as a loading control. Molecular-weight markers are shown in lanes 1 and 6.
B) Band analysis showing the calculated molecular weights and signal intensities of IFN-β and β-Tubulin for each condition.

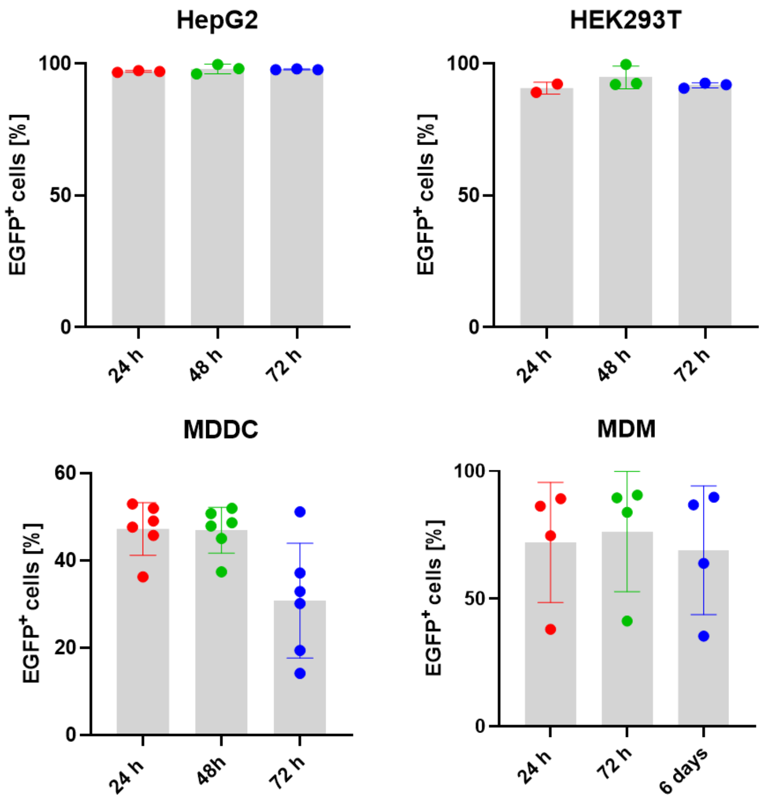

Figure S3. Transfection efficiency of the investigated cell types. Cells were transfected with EGFP-encoding mRNA under the respective experimental transfection conditions, and transfection efficiency was determined by flow cytometric quantification of EGFP-positive cells. Bars represent mean ± SD, and individual data points represent independent experiments.

Table S1: Inventory of antibodies used for flow cytometry analysis.

Marker	Fluorochrome	Clone	Vendor	Reference	Dilution
	Monocyte derived Dendritic cells Activation Panel				
**HLA-DR**	APC	G46-6	BD Biosciences	559866	1/ 33
**CD86**	V450	2331 (FUN-1)	BD Biosciences	560357	1/100
**CD80**	PE	L307.4	BD Biosciences	557227	1/33
**CD40**	PE-Cy™7	5C3	BD Biosciences	561215	1/100
**CD11c**	eFluor	3.9	Invitrogen	69-0116-42	1/100
**Viability Dye**	7-AAD	-	BD Biosciences	559925	1/33
	Monocyte derived Macrophages Polarization Panel				
**CD80**	PE	L307.4	BD Biosciences	557227	1/10
**CD86**	V450	2331 (FUN-1)	BD Biosciences	560357	1/40
**CD163**	PE-Cy™7	eBioGHI/61	Invitrogen	25-1639-42	1/40
**CD206**	APC	19.2	Invitrogen	17-2069-42	1/40
**Viability Dye**	7-AAD	-	BD Biosciences	559925	1/33
	Monocyte derived Dendritic Cells Co-Transfection Panel				

**CD80**	BUV395	2D10.4	Invitrogen	363-0809-42	1/20
**CD86**	V450	2331 (FUN-1)	BD Biosciences	560357	1/20
**HLA-DR**	BV605	G46-6	BD Biosciences	562845	1/20
**TNF-α**	BUV737	Mab11	Invitrogen	367-7349-42	1/20
**IFN-γ**	Alexa Fluor 488	4S.B3	BioLegend	502515	1/20
**IL-12p70**	PE	20C2	BD Biosciences	559325	1/5
**IL-6**	PE-Cy™7	MQ2-13A5	BioLegend	501120	1/20
**IL-8**	APC	E8N1	BioLegend	511410	1/20
**Viability Dye**	L/D Fix Near-IR	-	Invitrogen	L34975	1/1000

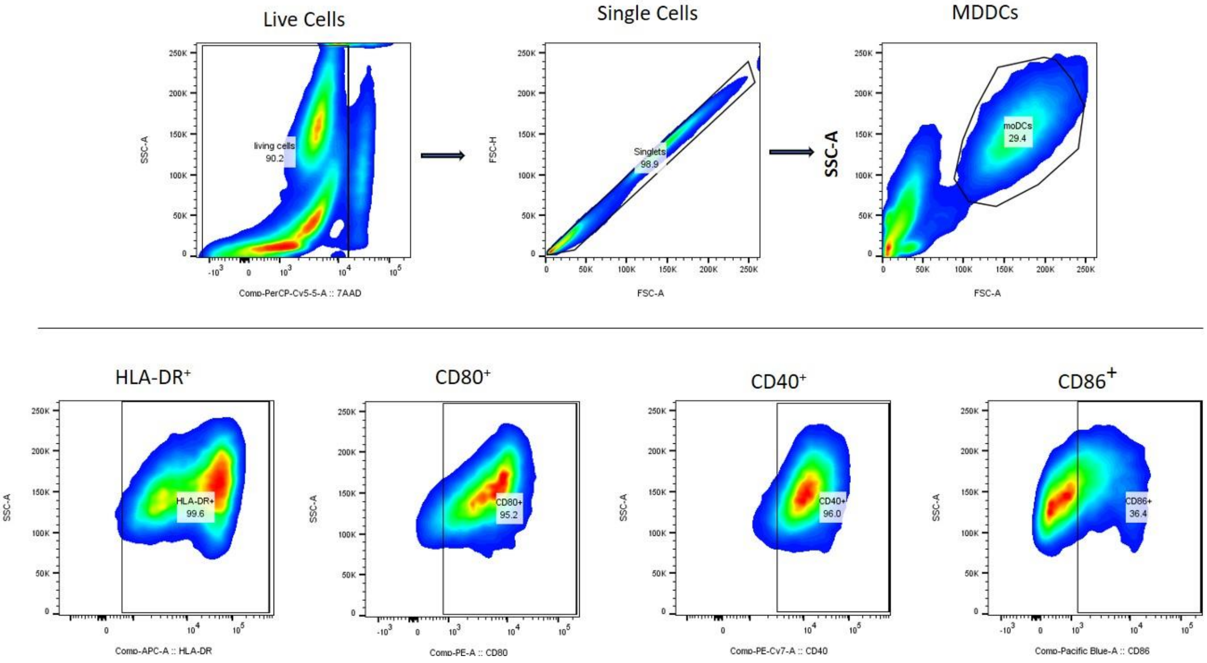

Figure S4. Gating strategy for MDDC activation. Representative flow cytometry dot plots showing the gating strategy used to assess MDDC activation based on the surface expression of HLA-DR, CD80, CD40 and CD86.

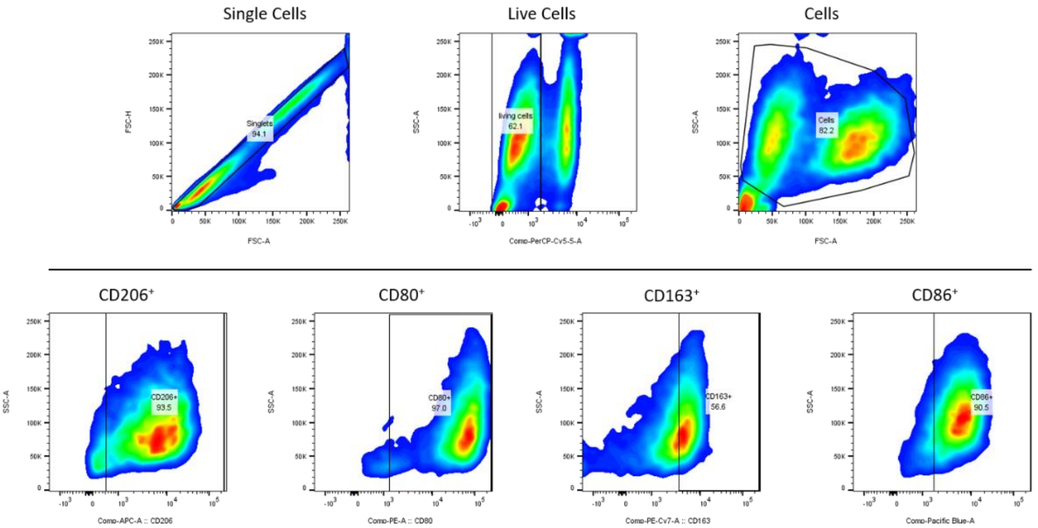

Figure S5. Gating strategy for MDM repolarization. Representative flow cytometry dot plots showing the gating strategy used to analyze MDMs polarized toward an M2 phenotype and subsequently treated with mRNA. CD206 and CD163 were used as M2 phenotypic markers, whereas CD80 and CD86 were used as M1 phenotypic markers.

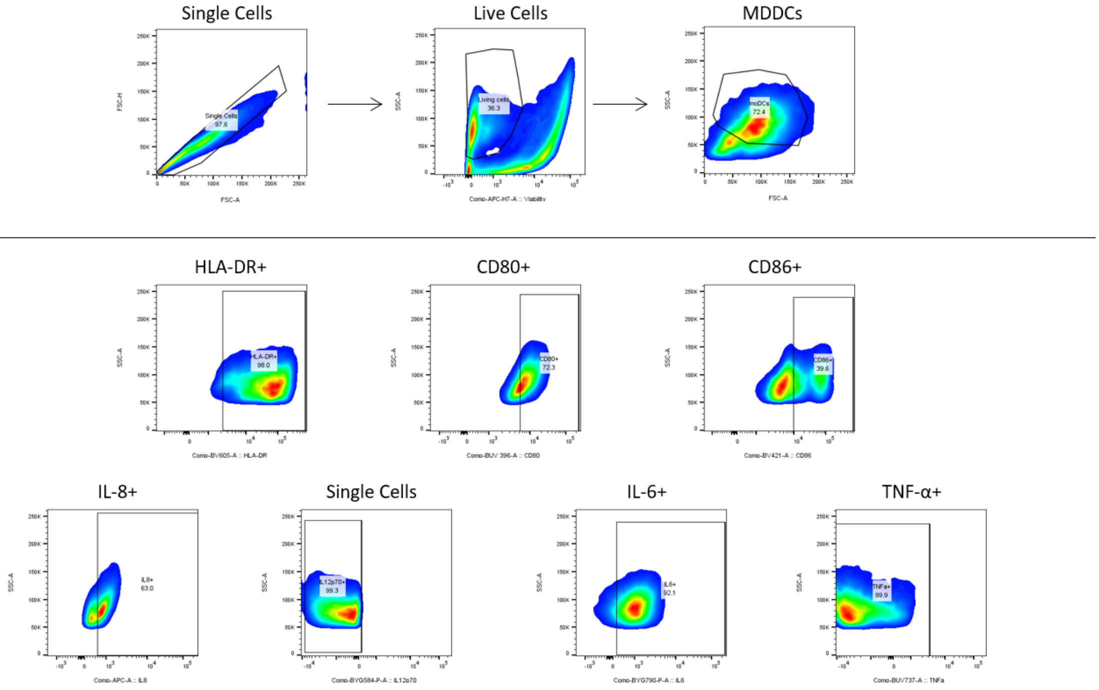

Figure S6. Gating strategy for surface and intracellular staining in co-transfected MDDCs. Representative flow cytometry dot plots showing the gating strategy used to assess the activation status of co-transfected MDDCs following adjuvant mRNA treatment. In addition to the surface activation markers HLA-DR, CD80 and CD86, intracellular staining was performed for the pro-inflammatory cytokines IL-8, IL-12p70, IL-6 and TNF-α.

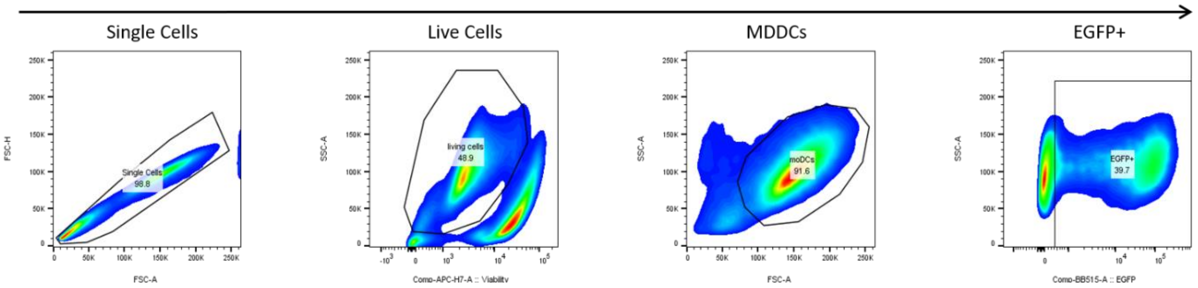

Figure S7. Gating Strategy for EGFP reporter mRNA expression in co-transfected MDDCs. Representative flow cytometry dot plots showing three gating strategy used to assess EGFP expression in MDDCs co-transfected with *EGFP* mRNA and stimulated with adjuvant mRNA.

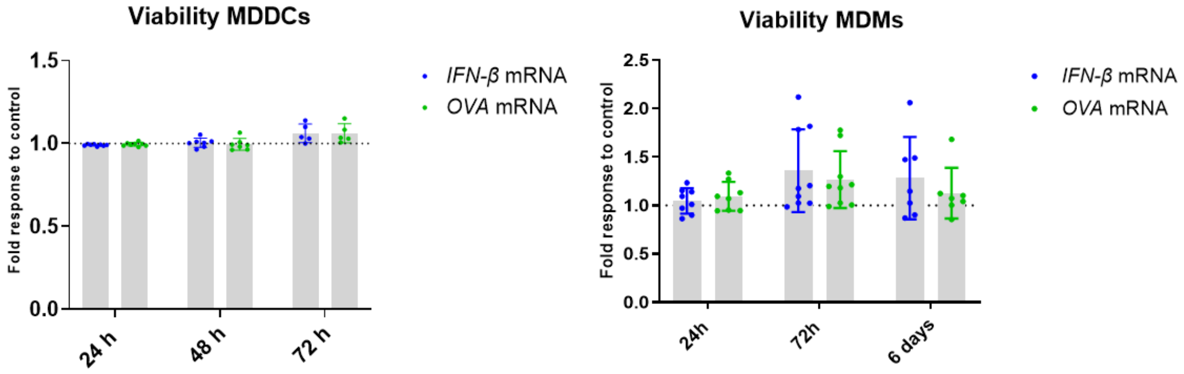

Figure S8. Assessment of viability in MDDCs as well as MDMs after mRNA transfection. Cell viability was evaluated by 7-AAD staining following transfection of MDDCs and MDMs with *IFN-β* mRNA or *OVA* mRNA. Viability remained stable in MDDCs after *IFN-β/OVA* mRNA transfection. IN MDMs, transfection with *OVA* mRNA and, to a greater extent, *IFN-β* mRNA was associated with increased viability. These results indicate that the mRNA constructs were not detrimental to MDDC or MDM viability.
